# SuMOS, a submerged microscope for observing substrates: Studying benthic activity in aquatic environments

**DOI:** 10.1016/j.ohx.2024.e00610

**Published:** 2024-11-15

**Authors:** Jens Wira, Allen R. Place

**Affiliations:** Institute of Marine and Environmental Technology, University of Maryland Center for Environmental Science, 701 E Pratt St, Baltimore, MD 21202, USA

**Keywords:** Submerged Camera System, Aquatic Substrates, Substrate Colonization

## Abstract

Described here is the construction of a low-cost, standalone underwater camera system designed for recording processes occurring on aquatic substrates. The Submerged Microscope for Observing Substrates (SuMOS) utilizes a Raspberry Pi Zero 2 W paired with a Raspberry Pi Camera Module v3 NoIR, with IR illumination for low-light situations. It features a waterproof housing inspired by the open-source PipeCam project, with enhancements for sealing and substrate mounting. The SuMOS system operates autonomously, capturing high-resolution images at fixed intervals under various lighting conditions. Tests in the Choptank River of Maryland demonstrated the system’s robustness in capturing patterns of amphipod activity under challenging optical conditions. This versatile tool offers a scalable solution for highly time-resolved, *in situ* studies of processes occurring at the interface of the aquatic/solid surface boundaries. The SuMOS provides significant advantages in cost, ease of deployment, and data collection.

## Graphical abstract

**Figure:**
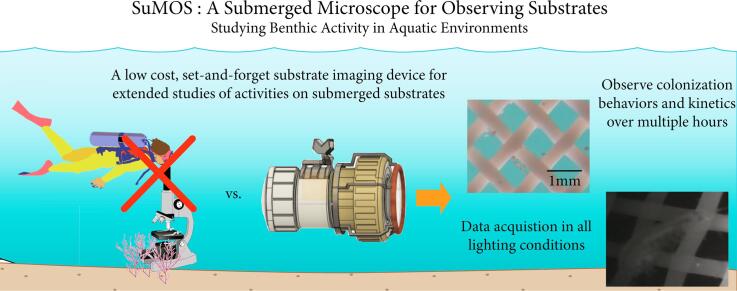

**Specifications table**

**Table:** 

Hardware name	Submerged Microscope for Observing Substrates (SuMOS)
Subject area	Biological sciences (e.g., microbiology and biochemistry)
Hardware type	• Imaging tools • Field measurements and sensors
Closest commercial analog	*No commercial analog is available.*
Open source license	*Creative Commons BY-SA*
Cost of hardware	*USD $250*
Source file repository	*https://doi.org/10.17605/OSF.IO/ZHBJF*

## 1 Hardware in context

Microscopic life in the marine environment has major impacts on our human environment and the global climate – from driving a planet-wide carbon cycle to harmful algal blooms (HABs) that affect local communities and fisheries [1]. Individual processes at this scale may seem inconsequential, but aggregation at the scale of the oceans gives them a massive impact [2]. Of these, organisms and processes occurring in the benthos are more diverse and myriad than those in the water column, and just as, if not more, important. Examples of such processes include transparent exopolymer deposition and biofilm initiation, benthic harmful algal blooms, and benthic invertebrate diel ecology [3], [4], [5], [6], [7]. However, studying these processes is often difficult due to logistical constraints deriving from high heterogeneity and an inability to easily integrate large areas or volumes as is possible with an unstructured water column [7], [8], [9], [10], [11].

The Submerged Microscope for Observing Substrates (SuMOS) is a standalone submerged camera device designed to study small-scale processes on underwater surfaces. The device provides an easily adaptable framework that can be used to study a variety of artificial substrates in a variety of aquatic environments. Substrates are mounted in front of an auto-focusing macro camera, and a timelapse video is taken of processes occurring on the substrate.

The external waterproof housing is modeled after the PipeCam, an open-source autonomous camera meant for similar time-lapses of aquatic environments. However, the SuMOS features additional optics for close-up imaging of fixed substrates, as well as an option for illuminating the subjects in pitch darkness [12]. The PipeCam enclosure style can be easily replicated using off-the-shelf parts available globally, and can be constructed with basic PVC supplies and tools. See (Table 1).

**Table 1:** Design Files Summary.

**Design file name**	**File type**	**Open source license**	**Location of the file**
Electronic Housings	.F3D CAD	*Creative Commons BY-SA*	*Available with the article*
Threaded Union Substrate Mount	.F3D CAD	*Creative Commons BY-SA*	*Available with the article*
PipeMount 90Degree	.F3D CAD	*Creative Commons BY-SA*	*Available with the article*
Union Wrench (Large)	.F3D CAD	*Creative Commons BY-SA*	*Available with the article*
Union Wrench (Small)	.F3D CAD	*Creative Commons BY-SA*	*Available with the article*

Studying benthic processes in-situ has been of interest for many years, where the earliest methods involved partially or fully submersible light microscopes, operated by divers or scientists to take observations [6], [7], [13]. However, as automated monitoring of phytoplankton in the pelagic region has taken off, advances in the study of aquatic benthic processes has lagged significantly [11]. For example, the study of biofouling on various materials is an area of active research, but in-situ rate measurements often offer limited temporal resolution based on human operator sampling schedules, which restricts the potential understanding of how different treatments or materials might affect the colonization process [9].

More recently, several groups have incorporated video or image recording capabilities into underwater microscopes for observing benthic processes *in-situ* [14], [15]. These devices are designed for high temporal resolution studies of microscopic processes, and as such are also designed to be handled directly by an operator for data collection. These underwater microscopes are meant to be used to study a large variety of natural substrates, which makes the manual setup and target acquisition critical to their use. These devices provide a more flexible experimental platform than the SuMOS, providing variable focusing, magnification, and study subject choices, since the operator is able to make decisions throughout the data acquisition process. However, the requirement for a diver operator greatly limits the time over which data can be continuously collected, which makes such designs insufficient for studying attachment and colonization kinetics for substrates, especially when diel patterns may be at play [16]. In addition, the inhomogeneity and stochastic nature of the marine/aquatic environment regularly demands large numbers of replicates for making statistically sound claims, which requires a more purpose-built design to facilitate experimental replication [17], [18].

The SuMOS opts for a “place-and-forget”, operator-free data acquisition process. Allowing the device to be fixed in place and subsequently collected for data retrieval greatly extends the possible study time, especially important for collecting data throughout the different parts of the diel period. The study substrate being mounted to the device housing within the autofocusing distance of the optics allows for a set-and-forget data collection process, where diver involvement is limited to placing and retrieving the device. The low cost and ease of deployment of the design also makes it possible for multiple devices to be built and deployed in parallel, in order to collect statistically significant sample sizes on realistic budgets. The device was designed as a framework with adaptability in mind, enabling a wide range of experiments to be conducted with different optical configurations or imaging schedules. See (Table 2).

**Table 2:** Waterproof Housing Bill of Materials.

**Designator**	**Component**	**Number**	**Cost per unit (USD)**	**Total cost (USD)**	**Source of materials**	**Notes**
PVC Pipe	*4″ Sch80 PVC*	16 cm	9	9		
PVC Union	4″ PVC Union	1	29.99	29.99	Amazon	
PVC Cap	4″ PVC Cap	1	12	12	Amazon	
Acrylic window	¼” clear acrylic panel	1	15	15	Amazon	
Securing Rods	5 mm Aluminium Extruded Rod	1	1	1	Amazon	
PLA filament	Polymaker PLA PRO Filament Black	1	25	25	Amazon	
TPU Filament	SpiderMaker SpiderFlex Matte Finish Flexible TPE(TPU)	1	5	5	Amazon	Cost is for weight used.

## 2 Hardware description

The main design objective of the SuMOS is to allow data collection with minimal operator interaction, which allows for data collection periods longer than a diver can be deployed for, to study processes that occur at different rates across the day-night cycle. As such, it focuses on recording the activity on fresh substrates mounted to the device, and is designed to be placed in natural environments without fine adjustments by a user. This deviates from other currently available hardware that requires operator control, but is able to observe natural substrates for short periods of time [14], [15].

The SuMOS is a fully standalone camera system that allows macro imaging of mounted substrates under various lighting conditions, and even in complete darkness. The device is designed to be assembled and deployed by operators in field conditions like dive boats with minimal tooling required, allowing ease of data collection from multiple locations or over multiple consecutive days. The device is assembled and activated outside of the water, and unlike previous underwater microscopes, human involvement in operation is only for the positioning and deployment of the device.

Using the SuMOS is simple: mount a study substrate, install a USB drive and power bank, seal the waterproof housing, deploy and retrieve the device, and the data is ready to be analyzed. The SuMOS captures a time-lapse video of the substrate at fixed intervals, as well as records the ambient light levels in lux, until the battery is depleted or removed. Both battery and USB drives are commercially available, and can be replaced while in the field, allowing for rapid redeployment of the device.

The external waterproof housing was inspired by PipeCam, an open-source autonomous camera, in which the main body is a length of capped PVC piping and a threaded PVC union with a clear plastic window for sealing and access [12]. The main change to the housing design is the use of a 3D-printed washer in the female end of the union that allows for the mounting of the study substrates, and for better distribution of the clamping force on the acrylic window. The study substrate is attached to the camera via a removable holder, and gross positioning to the focal plane is achieved by the use of spacers on the holder.

Inside the housing, electronics are housed in a 3D-printed assembly that is designed to slip into the waterproof 3D-printed housing. A compliant spacer is used in the assembly to apply compression of the assembly against the acrylic panel, as well as provide allowance for the use of PVC parts from different manufacturers, which tend to have some variability in design. The outer diameter of the assembly is designed to fit the interior diameter of a PVC Schedule 80 pipe, while the length of the assembly is adjustable by the use of spacers. All housing bodies are designed for ease of production with a hobbyist grade fused-deposition modelling 3D-printer, with geometry optimized to reduce the requirement for post-processing. See (Table 3).

**Table 3:** Internal Electronics Bill of Materials.

**Designator**	**Component**	**Number**	**Cost per unit (USD)**	**Total cost (USD)**	**Source of materials**	**Notes**
Raspberry Pi Zero 2 W	Raspberry Pi Zero 2 W	1	15	15	Adafruit	
SD Card	SanDisk 16 GB Ultra microSDHC UHS-I Memory Card	1	6	*6*	Amazon	
Camera Module	*Raspberry Pi Camera Module 3 NoIR − 12MP 75 Degree Infrared Lens*	1	25	25	Adafruit	
−Camera Ribbon Cable	Raspberry Pi Zero v1.3 Camera Cable	1	5.95	5.95	Adafruit	
TSL2591	Adafruit TSL2591 High Dynamic Range Digital Light Sensor − STEMMA QT	1	6.95	6.95	Amazon	
−Stemma QT connector	STEMMA QT / Qwiic JST SH 4-pin Cable with Premium Female Sockets	1	0.95	0.95	Adafruit	
IR LED	Adafruit Super-bright 5 mm IR LED	2	0.44	0.88	Amazon	
Indicator LED	CHANZON 3 mm LED Diode Lights	1	0.06	0.06	Amazon	Any 2–2.2 V LED is suitable.
RTC (Real time clock)	DS3231 Real Time Clock Module	1	3	3	Amazon	
Power Bank	Anker PowerCore 10,000 mAh Redux, Power Bank	1	35	35	Amazon	
Power Cable	Cable Matters Right Angle USB Cable	1	4	4	Amazon	Contains two cables, but only one is angled the right direction.
USB Drive	SanDisk 32 GB 3-Pack Ultra Fit USB 3.1 Flash Drive	1	6	*6*	Amazon	Other USB devices work, but the SuMOS was designed with this form factor in mind.
USB Data Cable	StarTech.com 5in Right Angle Micro USB to USB OTG Host Adapter M/F	1	3.86	3.86	Amazon	
Macro Lens	Leshareselect Professional 2 in 1 Lens Universal Clip 37 mm Mobile Phone Lens 0.45x 49uv Super Wide-Angle + Macro HD Lens for All Smartphones	2	7	14	Amazon	
+10 Diopter Lens	37 mm Closeup Filter Set	1	10	10	Amazon	
IR Resistor	56 Ω 1/4W Metal Film Resistor	1	0.01	0.01	Amazon	
Indicator Resistor	68 Ω 1/4W Metal Film	1	0.01	0.01	Amazon	

Imaging is driven by a Raspberry Pi Camera Module v3 NoIR and illuminated by two 940 nm IR LEDs in low light conditions. Ambient light levels are measured via a TSL2591 light sensor, and illumination is triggered when light levels fall below a user defined lux threshold. The device makes use of the Camera Module v3′s autofocusing capabilities coupled to macro lenses held before the lens, to provide the desired magnification and working distance while removing the need for manual fine focusing of the camera on to the imaged substrate. The Pi Camera allows for manual control of focus, which opens the possibility of data acquisition with focus stacking for future iterations, however the design choice was made to rely on a single autofocus due to battery and compute constraints.

Device control is achieved by a Python script running on a Raspberry Pi Zero 2 W single board computer. Data is saved locally on the Raspberry Pi’s local storage, and subsequently shuttled in chunks onto an attached USB device for data offload in order to reduce power draw over USB. Many of the software parameters, such as the imaging interval, the schedule for data offload from the Pi to the USB drive, and minimum lux threshold for IR illumination, can be controlled by modifying a configuration file loaded on the USB drive. This allows the device to carry out different experimental designs and conditions simply by editing a text file with a standard text editor. Without the configuration file, the device defaults to a set of sensible parameters that can be modified by the user but requires access to the Pi either via SSH or wired connection.

To summarize the advantages of our device:

• Operator-free method for studying colonization behavior of underwater substrates, enabling full day-night cycles to be captured.
• Ability to image underwater under different lighting conditions, including pitch darkness by means of IR illumination.
• Low cost and commercially available off-the-shelf parts, as well as ease of deployment and setup, allow for concurrent collection of multiple replicates on smaller budgets, enabling greater statistical power in experiments.
• Customizable framework for changing optical and data acquisition parameters provides flexibility in experimental design.

## 3 Design files summary

All files are Fusion 360 editable files with user parameters for editing dimensions that may vary for parts purchased from different vendors. All parts are in solid body format, and need to be exported as meshes before slicing for printing. The Electronic Housings file contains the internal framework for the SuMOS, in 4 bodies: the camera housing, Pi Housing, a spacer, and a cap. The Threaded Union Substrate Mount allows mounting of substrates to the union, with the thread pitch, and housing diameter as parameters. The PipeMount 90Degree is for mounting the SuMOS to a pipe by means of hose clamps, with the size of the pipe as an editable parameter. The Union Wrenches are meant to provide torque for sealing or unsealing the SuMOS for deployment, and the size and shape of the union to be torqued is specifiable as parameters.

## 4 Bill of materials summary

The total cost of the components at time of writing did not exceed USD$250.

## 5 Build instructions

### 5.1 External waterproof enclosure

The construction of the enclosure is heavily inspired by the PipeCam project [12], with the key deviation being the use of the substrate mounting flange. A more detailed build instruction can be found at the source, but briefly:

1. Cut the Schedule 80 4″ PVC pipe to 6.5″.
2. Using the appropriate primers and PVC cement, attach the PVC cap to one end, and the side of the union with the O-ring groove to the other. An example of the assembled housing can be seen in Fig. 1b.

**Fig. 1:**
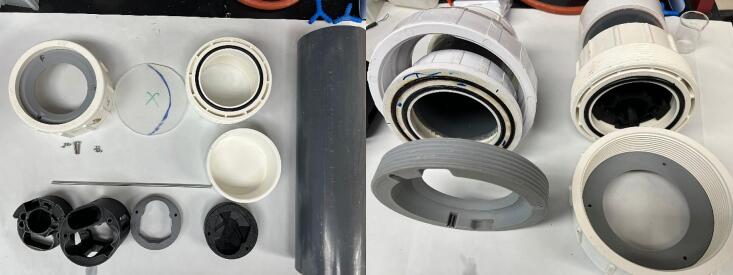
(a, left) Parts for the waterproof enclosure and internal electronic housing, note the O-ring mounting groove on the union fitting. (b, right) Two completed housings using the two configurations of commercially available PVC Unions, as well as substrate mounting flanges. The mounting flange is fitted in the collar of the right one. Note: Different union manufacturers have different designs. Specifically, the O-ring may be housed on different sides of the union in relation to the threading, which dictates the design of the substrate mounting flange. Depending on availability and cost, users may run into one of two design options. The first (left of Fig. 1b) has the threaded fitting on the opposite piece of the O-ring, and the 3D printed mounting flange is threaded and secured against the threaded union collar to provide compression between the acrylic panel and O-ring for sealing. In our testing, the mounting flange could be printed with 4–5 walls and 30 % infill, since the force was spread across the threads. The second (right of Fig. 1b) has the threaded fitting on the same piece as the O-ring groove, and the threaded collar alone is sufficient to provide compression for sealing. In this configuration, the mounting flange is inserted into the collar, and will be the mating interface with the acrylic panel. The mounting flange had to be printed fully solid since the compressive force was concentrated on the top and bottom surfaces. The .f3d design files for both options are provided but may require modifications for the specific thread specifications of different manufacturers and are provided as editable parameters.
3. Cut the 1/4″ acrylic panel to fit over the O-rings, and in the union. This can be done with a bandsaw or router as available.
4. 3D-print the appropriate mounting flange for the union type.

The sealing of the external waterproof housing can be tested by pressurizing it internally. A valve drilled into a spare acrylic panel can be used to provide means to pressurize the housing upon sealing, and can be swapped between multiple housings to be tested. An important safety note is to minimize the amount of compressible air inside the system to reduce explosion risk at high pressures, so if hydrostatic testing facilities are not available, filling the housing with water before pressurizing with air reduces the risk significantly.

### 5.2 Internal electronics housing

1. 3D-print the Camera and Access Housing, RPi Zero2 Mounting Piece, and Scalloped End Cap in a rigid filament, and the compliant spacer in a flexible elastomer.
2. Insert two heatset inserts into the top face of the RPi Zero2 Mounting Piece for securing the Camera and Access housing.
3. Cut the 5 mm Aluminium rods to length, and slide the assembly into place as indicated in Fig. 2.

**Fig. 2:**
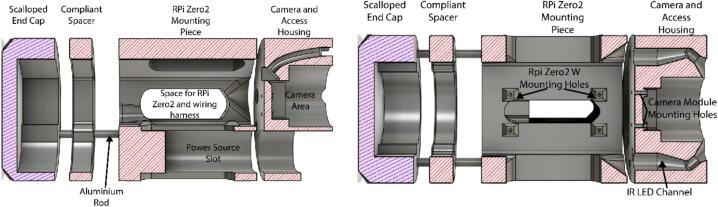
Section view schematics for 3D printed internal electronic housing.
4. Using hot glue or epoxy, attach the Scalloped End Cap to the inside of the waterproof housing cap, and mark on the external housing where “up” is for the camera. This ensures the internal housings do not rotate freely, and the device can be mounted in the proper orientation.
5. If necessary, measure any excess space in the external housing after insertion of the electronic housings, and 3D-print an additional spacer in a rigid filament to fill the space. In practice, this is a simple method to account for variation in the external housing lengths.

### 5.3 Electronics assembly

1. Connect the ribbon cable to Raspberry Pi Camera Module, the Stemma QT connector to the TSL2591, and solder wires to the leads of the LEDs.
2. Solder a 56 Ω resistor to each IR LED, and a 68 Ω to the 2.2 V red indicator LED
3. Remove jumper housing from female jumpers, and pass wires through appropriate channels. Fig. 3 provides a visual guide for where each cable goes.

**Fig. 3:**
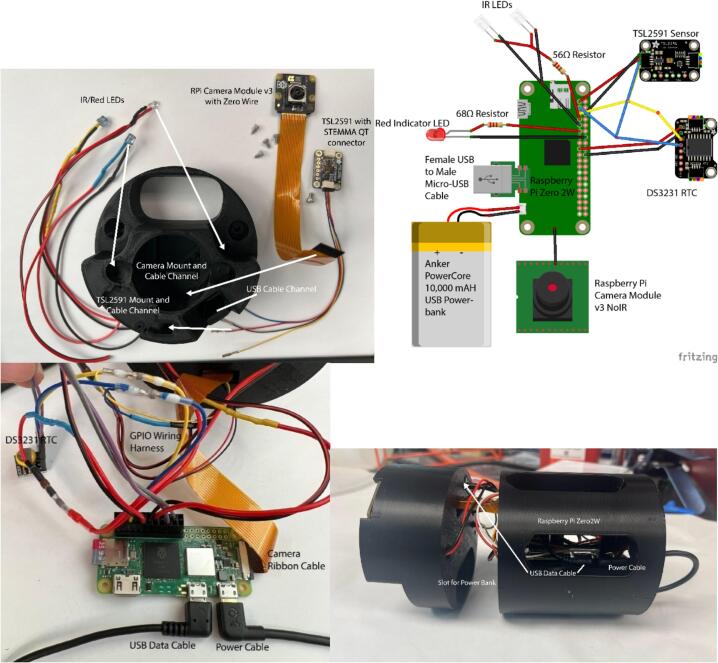
(a) Unassembled view of parts for front facing camera mount, showing appropriate mounting areas and channels for different wires. (b)Wiring diagram sketch. (c) Electronics connected and labeled, showing appropriate connections to the Raspberry Pi board. (d) Electronics assembled in housing, showing location of cabling and battery bank.
4. Crimp female jumper heads to the end of the LED leads.
5. Attach the camera and TSL2591 to the Camera Housing with M2 hex screws. Insert the macro-lenses into the channel above the camera, and hot glue them in place.
6. Connect the power, ground, data and clock lines to the DS3231 RTC, and splice the data and clock lines to the same lines as the TSL2591 so they share the same I2C bus.
7. Insert jumper leads into the plastic housings, and connect these to the RPi Zero 2 W, following the wiring diagram in Fig. 3b. For added security, breadboard jumper housings can be used as to provide more surface area for mounting. The assembly in Fig. 3c uses a 6x2 and 5x2 set for connection.
8. Connect the camera ribbon cable to the CSI port, the male-male microUSB – USB cable to the power port, and the male–female microUSB – USB cable to the USB port.
9. Insert the USB end of the power cable through the channel in the rear of the RPi Zero 2 W Mounting Piece, taking care that the orientation matches that of the power bank. Secure with hot glue.
10. Pass the female end of the data cable through the data cable channel in the Camera Housing.
11. Attach the RPi Zero 2 W to the Mounting Piece with M2.5 screws, through the holes in the housing.
12. After loading the OS onto the microSD card, insert the microSD card into the RPi Zero 2 W, then using the appropriately sized machine screws, attach the two housings together.

The housing was designed in two pieces to facilitate assembly as well as production, with the use of the heat-set inserts and machine screws to join them into a single unit for security. In our prototyping, both lever nut joins and solder joins were suitable, with the lever nuts being easier to use but slightly bulkier in the final assembly. Use of hot-glue on the GPIO jumpers also provided additional security in case of vibrations/movement when deployed. Fig. 3c serves as a useful reference for connections to the Raspberry Pi board, and Fig. 3d for the final assembly.

### 5.4 Software installation

1. Using the provided Raspberry Pi OS imager (https://www.raspberrypi.com/software/) install the legacy lite 64-bit OS (Bullseye) onto an SD card of at least 8 GB, with the username “sumos”. It is also convenient at this step to provide details of an available WiFi network in the configuration.
2. From the associated SuMOS repository, download the python and shell scripts located in the “Install Scripts” folder. Transfer them onto the SD card into the home directory, or via SCP after initialization.
3. Insert the SD card, into the RPi Zero 2 W, and power it on. Wait for the device to initialize.
4. Find the IP address of the device over the local network, and connect to it over SSH.
5. With the install.sh script in the home directory, run “sudo bash install.sh”. The device will carry out the installation and require a restart to determine that everything has successfully been set up.

After the restart, the SuMOS can be fully assembled and is ready to be used. The install.sh script installs the prerequisite packages, sets up the GPIO pins and activates the I2C channel, and creates a system service that calls the data acquisition script at startup. An included script “streamControlLED.py” is included to test the device hardware and attachments, and also provides a livestream of the camera over a LAN webserver.

## 6 Operation instructions

The SuMOS was designed for ease of deployment and replacement in the field, and as such operational steps are few once the device is fully assembled. The device simply needs to be initialized, sealed, and deployed.

1. Device initialization

Initializing the SuMOS is as simple as inserting a USB storage device and USB power bank into the appropriate ports. The red indicator LED will turn on when the system boots successfully. The device then runs through an initial startup check, during which if any step of the regular imaging and data transfer workflow fails, the device enters an error state, and the red LED will persistently flash in a pattern that depends on which subsystem triggered an error. Should the startup sequence finish successfully, the red LED will turn off, and the device will continue to record data until the battery is removed or runs dry.

A set of default data acquisition settings is stored on the device, and can be changed in the main capture script (captureScript.sh) over a ssh connection, or when first loaded. A configuration file can also be placed in the main directory of the USB device that is used to set the recording parameters on a per run basis without the need for access to the OS of the SuMOS. The configuration file is provided in the source repository (divePiSettings.conf).

2. Sealing waterproof housing

Upon successful powering up of the device, the outer housing needs to be sealed. The acrylic panel is placed over the top of the union, and the 3D-printed mounting flange is then screwed in to seal the device. Care should be taken to keep the O-rings clean and free from debris, as well as to lubricate the O-rings with silicone grease. Additional torque for sealing the PVC union is provided by use of pipe wrenches. A set of 3D model files for wrenches that fit the exact unions used are provided, and the parameterized.f3d files are provided for modifying the wrench to fit other unions.

3. SuMOS deployment

**Fig. 4:**
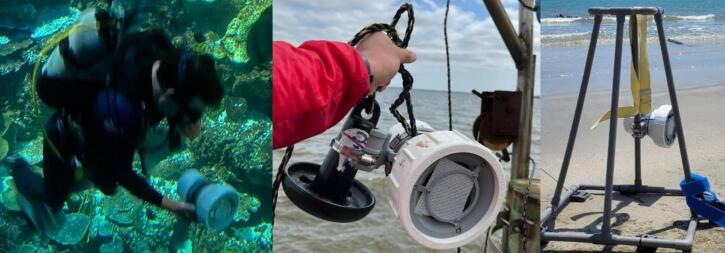
Examples of how the SuMOS can be deployed. Left to right, in calm protected waters with a dive weight, attached to a mushroom anchor off a pier, or on a weighted transect sampler.

The SuMOS can be deployed in a variety of manners dependent on the environmental conditions and requirements. Two mounts are provided for mounting the device vertically or horizontally via the use of hose clamps onto available hardware, depending on field conditions and requirements (Fig. 4).

## 7 Validation and characterization

The SuMOS in the described configuration provides a field of view of 18x10 mm, with a resolution of 4608x2592 pixels (12 MP), resulting in a pixel pitch of 3.9 μm/pixel, at a working distance of approximately 10 mm from camera to substrate. At this short working distance, the LEDs were not able to uniformly illuminate the imaging area, but sufficient light would reach the field of view to enable imaging. Operating at an imaging schedule of 1 photo/min, the camera was capable of up to 30 h of non-stop imaging, exceeding the 24 h required for a full day-night cycle.

Multiple waterproof housings were constructed and tested for their sealing. All were able to hold 50PSI of pressure for at least 24 h, which would indicate a static waterproof rating of at least 50 m. Although not a direct test of watertightness due to the different direction of the pressure delta from field conditions, both this testing and the pressure ratings of the different joins exceed the depths which divers would usually be at. The waterproof housing was also tested at a depth of 15 m in calm protected seawater, without any evidence of water ingress.

**Fig. 5:**
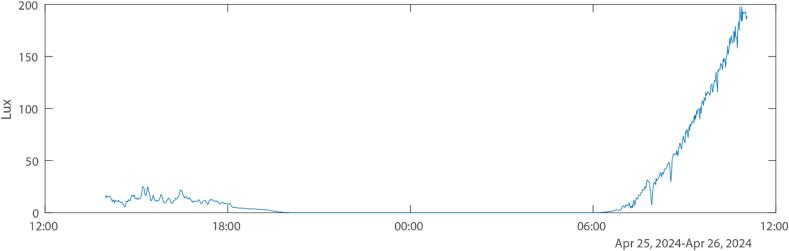
Graph of measured light levels across the 24-hourr deployment, showing strong variability in lighting during the day periods.

**Fig. 6:**
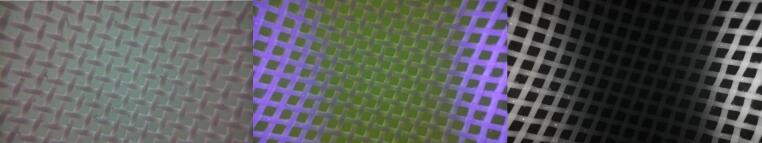
Example images from SuMOS deployment showing images over decreasing light levels, with the final image in pitch darkness.

To observe in-field performance, the SuMOS was tested over a period of 24 h at the PhytoChop Observatory, at Horn Point Laboratory, Cambridge MD. Deployment was carried out by mounting the SuMOS onto a mushroom anchor, and lowering the device off a pier to rest on the riverbed of the Choptank River at a depth of about 2 m. This provided challenging optical conditions for imaging, due to moderate current and constant sediment resuspension, as well as overcast conditions on the first day. The light levels as measured by the SuMOS can be seen in Fig. 5. The study substrate was a mounted 1 mm fiberglass mesh, and the device was able to keep focus on the mesh for the entire deployment across the differing conditions, as seen in Fig. 6.

The resulting data is a timelapse video of what occurred on the substrates, and as such, processing is highly dependent on the type of experiment that is being carried out. Here we describe an exemplar method of data analysis that takes advantage of regular image processing techniques to filter out amphipods and other large objects of interest in the field of view for manual annotation.

**Fig. 7:**
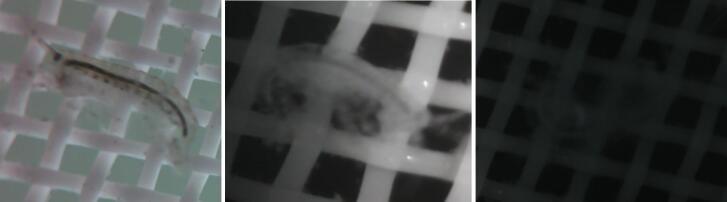
Example of filtered amphipod images across multiple light levels, showing the utility of the segmentation method for picking out images of interest.

The RGB image is first converted to the L*A*B space, and a flatfield correction is applied to the L channel. The L channel is then subtracted from a moving median window. The magnitude of the difference is thresholded to determine regions with significant departures from the average image, and also subjected to a Canny edge filter to connect adjacent regions together, important for slightly transparent samples. The two binary masks are combined, and the resulting blobs are morphologically opened to remove small blobs. Many amphipods, as well as regions with large organic matter attachment were successfully extracted. For our purposes, this resulted in a substantially smaller data set in which amphipods could be manually annotated. With more data, the annotations can also be used to train CNN recognition models for a more objective method of analysis, if desired. Examples of the amphipods detected are shown across different light levels (Fig. 7).

**Fig. 8:**
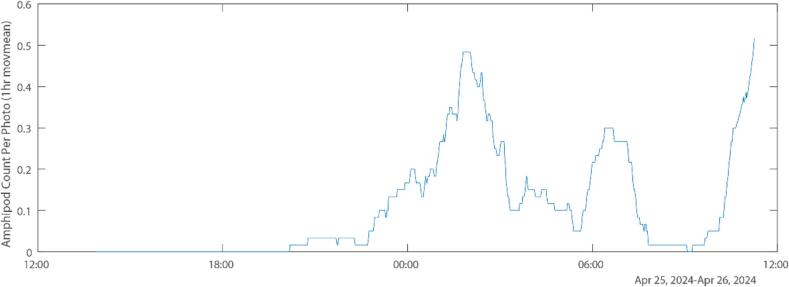
Plot of amphipods recorded per photo, smoothed with a moving mean with a 1 h window. A total of 85 photos out of 900 contained amphipods.

The results of the annotation were analyzed to demonstrate the utility of the SuMOS, and show the presence of amphipods after midnight, only approx. 10 h of deployment (Fig. 8). This matches closely with observations made on related benthic harpacticoid copepods, in that colonization of similar artificial mesh screens began only at midnight [19]. That experiment involved significant labor investment, requiring operators to deploy and collect screens throughout the night, as well as sample processing and counting of attached copepods. Meanwhile, the utility of the SuMOS is clearly demonstrated here, involving a single deployment and retrieval. It should be noted, the small sample size used here means that it is advisable that more deployments be carried out to make ecological determinations as to the diel patterns of the amphipod. Nevertheless, the utility of the SuMOS for studying biological activity in-situ, coupled with its ease of use and deployment, cannot be overstated.

## CRediT authorship contribution statement

**Jens Wira:** Writing – review & editing, Writing – original draft, Validation, Software, Resources, Methodology, Investigation, Funding acquisition, Conceptualization. **Allen R. Place:** Writing – review & editing, Supervision, Resources, Funding acquisition, Conceptualization.

## Declaration of competing interest

The authors declare that they have no known competing financial interests or personal relationships that could have appeared to influence the work reported in this paper.

## References

[1] Falkowski P. (2012). Ocean science: the power of plankton. Nature.

[2] Levin S.A. (1992). The problem of pattern and scale in ecology: the Robert H MacArthur Award Lecture. Ecology.

[3] Institute of Marine Sciences (CSIC), Barcelona, E. Berdalet, P. Tester, M. Chinain, S. Fraga, R. Lemée, W. Litaker, A. Penna, G. Usup, M. Vila, A. Zingone, Harmful algal blooms in benthic systems: recent progress and future research, Oceanog. 30 (2017) 36–45. https://doi.org/10.5670/oceanog.2017.108.

[4] Passow U. (2002). Transparent exopolymer particles (TEP) in aquatic environments. Prog. Oceanogr..

[5] Hotos G., Bekiari V. (2023). Absorption spectra as predictors of algal biomass and pigment content of the cultured microalgae Amphidinium carterae, Isochrysis galbana, Nephroselmis sp., and Anabaena sp.. Int. J. Plant Biol..

[6] Neushul M. (1972). Underwater microscopy with an encased incident‐light dipping‐cone microscope*. J. Microsc..

[7] Kennelly S.J., Underwood A.J. (1984). Underwater microscopic sampling of a sublittoral kelp community. J. Exp. Mar. Biol. Ecol..

[8] C.M. Lalli, T.R. Parsons, CHAPTER 7 - BENTHOS, in: C.M. Lalli, T.R. Parsons (Eds.), Biological oceanography: an introduction (Second Edition), Butterworth-Heinemann, Oxford, 1997: pp. 177–195. https://doi.org/10.1016/B978-075063384-0/50063-3.

[9] Delgado A., Power S., Richards C., Daly P., Briciu-Burghina C., Delauré Y., Regan F. (2023). Establishment of an antifouling performance index derived from the assessment of biofouling on typical marine sensor materials. Sci. Total Environ..

[10] Lobel P.S., Anderson D.M., Durand-Clement M. (1988). Assessment of ciguatera dinoflagellate populations: sample variability and algal substrate selection. Biol. Bull..

[11] Erickson J.S., Hashemi N., Sullivan J.M., Weidemann A.D., Ligler F.S. (2012). In situ phytoplankton analysis: there’s plenty of room at the bottom. Anal. Chem..

[12] PipeCam: Low-Cost Autonomous Underwater Camera, (n.d.). https://hackaday.io/project/21222-pipecam-low-cost-autonomous-underwater-camera (accessed October 30, 2023).

[13] Staley J.T. (1971). Growth rates of algae determined in situ using an immersed microscope1. J. Phycol..

[14] Mullen A.D., Treibitz T., Roberts P.L.D., Kelly E.L.A., Horwitz R., Smith J.E., Jaffe J.S. (2016). Underwater microscopy for in situ studies of benthic ecosystems. Nat. Commun..

[15] Shahani K., Song H., Mehdi S.R., Sharma A., Tunio G., Qureshi J., Kalhoro N., Khaskheli N. (2021). Design and testing of an underwater microscope with variable objective lens for the study of benthic communities. J. Marine. Sci. Appl..

[16] A. Moriera, P.A. Tester, Methods for sampling benthic microalgae, in: Guide for Designing and Implementing a Plan to Monitor Toxin-Producing Microalgae, 2nd ed., Intergovernmental Oceanographic Commission (IOC) of UNESCO and International Atomic Energy Agency, 2016: pp. 19–28. https://unesdoc.unesco.org/in/documentViewer.xhtml?v=2.1.196&id=p::usmarcdef_0000214510_eng&file=/in/rest/annotationSVC/DownloadWatermarkedAttachment/attach_import_d44c9418-7aa9-4f4d-9280-7ca3e866bcce%3F_%3D214510eng.pdf&locale=en&multi=true&ark=/ark:/48223/pf0000214510_eng/PDF/214510eng.pdf#%5B%7B%22num%22%3A26%2C%22gen%22%3A0%7D%2C%7B%22name%22%3A%22XYZ%22%7D%2Cnull%2Cnull%2C0%5D (accessed June 28, 2023).

[17] F. Taylor, M. Gustavson, An underwater survey of the organism chiefly responsible for “ciguatera” fish poisoning in the eastern Caribbean region: the benthic dinoflagellate Gambierdiscus toxicus, in: Proceedings of the 7th. International Diving Science Symposium. Padova, Italy. CMAS, University of Padua, Italy, 1986: pp. 95–111.

[18] Tester P.A., Litaker R.W., Soler-Onís E., Fernández-Zabala J., Berdalet E. (2022). Using artificial substrates to quantify Gambierdiscus and other toxic benthic dinoflagellates for monitoring purposes. Harmful Algae.

[19] Hauspie R., Polk Ph. (1973). Swimming behaviour patterns in certain benthic harpacticoids. Crustaceana.

